# Functional Outcomes of Brolucizumab-Induced Intraocular Inflammation Involving the Posterior Segment—A Meta-Analysis and Systematic Review

**DOI:** 10.3390/jcm12144671

**Published:** 2023-07-14

**Authors:** Justus G. Garweg, Judith Keiper, Isabel B. Pfister, Christin Schild

**Affiliations:** 1 Swiss Eye Institute and Clinic for Vitreoretinal Disease, Berner Augenklinik, 3007 Bern, Switzerlandisabel.pfister@augenklinik-bern.ch (I.B.P.); christin.schild@augenklinik-bern.ch (C.S.); 2 Department of Ophthalmology, Inselspital (Bern University Hospital), University of Bern, 3010 Bern, Switzerland

**Keywords:** neovascular age-related macular degeneration, brolucizumab, intraocular inflammation, uveitis, retinal vasculitis, retinal vascular occlusion, treatment outcome

## Abstract

Early poor outcomes of intraocular inflammation (IOI) after intravitreal brolucizumab (IVB) have negatively affected the use of brolucizumab in clinical routine. We wished to identify factors related to the treatment details of IOI involving the posterior segment resulting from IVB for neovascular AMD (nAMD), if these were reported in detail. Articles were retrieved from PubMed, Scopus, ClinicalTrials, and CENTRAL using the following search terms: <Brolucizumab> AND <AMD> AND <intraocular inflammation>. The risk of bias was rated using the JBI Critical Appraisal Tool. We included 31 reports (41 patients and 46 eyes). Patients were 75.9 ± 8.5 years, and 58.5% were female. IOI occurred 41.7 ± 37.5 (median 37.0) days after treatment initiation with 2.0 ± 1.3 (1–6) IVB injections. A mean change in visual acuity of −14.6 ± 21.0 (median −6.5) letters was reported. The mean time from first IOI signs to the initiation of any anti-inflammatory treatment was 3.3 ± 6.2 days, with 63% of the patients receiving systemic corticosteroids as standard treatment. Finally, a period effect was observed, with a change in visual acuity of −25.3 ± 27.1 and −2.6 ± 7.3 letters in the chronologically first and last third, respectively, of treated eyes (effect size: r = 0.71; *p* = 0.006). Functional outcomes markedly improved with increasing experience in managing IOI.

## 1. Introduction

The introduction of anti-vascular endothelial growth factor (VEGF) agents for treating exudative or neovascular age-related macular degeneration (nAMD) in 2006 and diabetic maculopathy (DME) in 2010 has dramatically improved the treatment outcomes of these previously blinding diseases [1]. However, existing treatment options (bevacizumab, ranibizumab, and aflibercept) have a considerable rate of incomplete therapeutic responses related to persistent retinal fluid, a high treatment demand, and an unacceptable treatment burden. This incomplete treatment response leads to long-term preventable vision loss as well as the progression of the underlying vascular pathology in AMD [2,3,4,5,6,7] and DME [8,9,10,11]. To some extent, optimizing the treatment strategies has improved the outcomes in nAMD [12,13,14], but not in DME [15]. 

A major concern related to long-term anti-VEGF treatment for nAMD, but not for DME, is the occurrence of macular geographic atrophy (MA). Permanent VEGF suppression may contribute to choroidal thinning; contrastingly, persistent subretinal fluid may exert protective effects. Treatment with anti-VEGF agents increases the overall risk of MA [16,17], which partly explains why up to 80% of patients with nAMD often terminate their injection therapy [1].

Brolucizumab is a newer, stronger—regarding its effects on retinal fluid—and longer-acting anti-VEGF agent, which allows a lower rate of incomplete treatment, a reduction in the treatment burden, and an extension of the mean treatment intervals, as demonstrated in two large-scale phase 3 randomized clinical trials (HARRIER and HAWK) [18] as well as early real-life experience [19,20,21,22,23,24,25,26,27,28,29,30,31]. Although it has promising therapeutic efficacy, it has been reported to result in intraocular inflammation (IOI) and even severe vision loss [22,32,33,34,35,36,37,38]. This has considerably limited its use in real-life clinical settings, which is worsened by the lack of clear guidelines for the management of IOI [39]. Nonetheless, several groups have published guidelines based on expert opinions [13,40,41,42,43,44]. Accordingly, there has been renewed interest in brolucizumab given its therapeutic efficacy [45,46], duration of action, and cost efficacy [23,34,47,48].

Brolucizumab-induced IOI without retinal vascular changes has a mild course in >50% of cases upon the permanent discontinuation of brolucizumab [32,37,49]. The current literature regarding brolucizumab-induced IOI involving the posterior segment is limited to the risk factors for severe vision loss in the absence of early diagnosis and aggressive treatment [38,50]; however, the clinical profile and treatment outcomes of this acute immune-mediated disease remain unclear [51]. Accordingly, we aimed to perform a systematic review and meta-analysis of the clinical profile, time to diagnosis, treatment strategy, and treatment outcomes of brolucizumab-induced IOI involving the posterior segment.

## 2. Patients and Methods

We performed a systematic literature search for articles published until 3 November 2022 on the PubMed, Scopus, ClinicalTrials, and CENTRAL databases using the following key terms: <Brolucizumab OR Beovu> AND <AMD OR macular degeneration OR macula> AND <inflammation OR vasculitis OR occlusion OR uveitis>, without any exclusion criterion, based on the PRISMA (Preferred Reporting Items for Systematic Reviews and Meta-Analyses) guidelines. The full search strategies are presented in the Supplementary Materials. The reference lists of published reports, meta-analyses, and reviews were screened for suitable original articles. We included all articles related to brolucizumab-induced IOI and its treatment outcomes on a single-patient basis that were written in English, French, or German. We included all articles reporting on cases with age-related macular degeneration (P), which were treated with brolucizumab (I), compared by their visual outcome (C), reported on following intraocular inflammations of the posterior segment, and published as single cases or case series (S) to obtain as much detailed information as possible on therapeutic factors explaining the outcomes of each case according to PICOS criteria. We accepted retrospective and prospective studies as far as they provided a detailed description of the clinical presentation, course, and treatment for single eyes. The exclusion criteria for articles were as follows: summarizing outcomes without referring to single patients; referring to cases without the involvement of the posterior segment; reviews and meta-analyses that summarized previously reported cases; and editorial notes that did not report new observations. All references were managed using the Endnote software. We performed automatic and manual screening for duplicates. Based on the need for a specific search for single patient data in order to address the study questions, we waived study registration. 

Two independent researchers screened all the articles based on the inclusion criteria, with discrepancies being resolved in discussions. One author extracted the data from all suitable articles in a two-step process. First, all necessary information based on a coding sheet draft was entered, with additional categories being added as appropriate. Second, missing data were specifically searched for in the research papers. All data entries were confirmed by the first author. 

We extracted the following information: general information including identification (first author and publication year), demographic characteristics (age, sex, country, and general and ocular history), timing and type of pretreatment with other anti-VEGF agents, confirmatory diagnostic tests, treatment of nAMD until the IOI diagnosis, time until IOI treatment, IOI treatment, and treatment outcomes. 

We only included peer-reviewed journals to ensure an appropriate level of methodological robustness. Generally, inherent bias could not be avoided given the small number of observations per paper and the multiple confounding factors, including time until IOI diagnosis, treatment initiation, treatment route, and therapy duration. The risk of bias was assessed using the JBI Critical Appraisal Checklist for Case Series, which was found to be medium to high due to missing and inconsistent longitudinal outcome reports (see Supplemental Table S1) [52]. In general, case reports were rated higher for risk of bias than case series since they met less criteria. One has to take into account that the assessment tool was created for case series rather than reports.

### Statistical Analyses

Descriptive statistics were applied since we sought to summarize the existing evidence without a control group. Since the data were not normally distributed, we performed group comparisons using the Mann–Whitney U test. For significant results, we also reported the effect size using the Pearson’s correlation coefficient r. All visual acuity results were transformed into early treatment of diabetic retinopathy score (ETDRS) values, where a Snellen decimal best-corrected visual acuity (BCVA) of 1.0 was defined as 85 ETDRS letters. Statistical significance was set at *p* < 0.05. Statistical analyses were performed using the SPSS software package 28.0.1 (SPSS, Inc., Chicago, IL, USA) and R (version 3.2.4; R: A language and environment for statistical computing, R Foundation for Statistical Computing, Vienna, Austria, 2016).

## 3. Results

**Figure 1:**
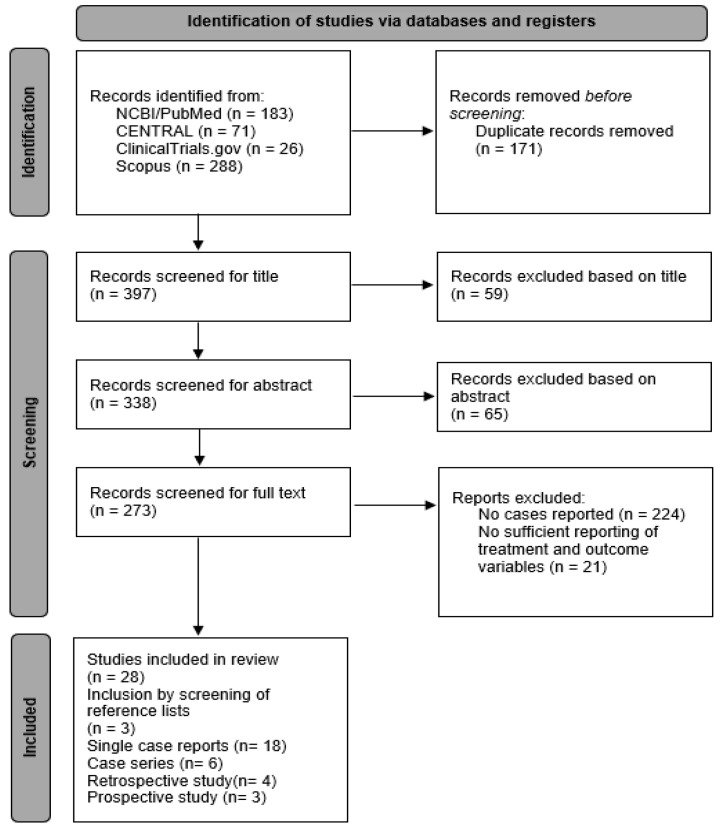
PRISMA flow diagram.

Initially, 568 articles were screened; among them, 31 studies (41 patients; 46 eyes; Figure 1) met the inclusion criteria [21,22,23,25,27,35,36,50,53,54,55,56,57,58,59,60,61,62,63,64,65,66,67,68,69,70,71,72,73,74,75]. A full list of the included and excluded articles, as well as the extracted data and template, can be obtained upon request from the first author. Figure 1 shows a prismatic flow diagram of the included studies.

### 3.1. Demographics

We included 41 patients (46 eyes) in the analysis (Table 1). Notably, there were no regular descriptions of the baseline clinical findings, evolution of the disease, diagnostic methods, or treatment outcomes. Baseline visual acuity (before the initiation of intravitreal brolucizumab treatment) and evolution of visual acuity were reported in 44 (95.7%) and 41 (89.1%) eyes, respectively.

The mean age of patients (n = 38; 3 missing patients) was 75.9 ± 8.5 (52–94) years; further, 58.5% (24/38) of the patients were females. Until the occurrence of IOI, the eyes had received one to six (2.0 ± 1.3; median 2.0; IQR 1.0–2.0) intravitreal brolucizumab injections (n = 45). The duration of brolucizumab treatment until IOI development was 41.7 ± 37.5 (median 37.0; IQR 14.0–54.3) days. Moreover, 20 and 25 eyes received only one and two to six brolucizumab injections, respectively (one eye was not reported). 

### 3.2. Evolution of Visual Acuity 

Table 2A shows the evolution of BCVA. The change in visual acuity from brolucizumab initiation until the last follow up (18–56 days after IOI onset; n = 35) ranged from −66 to +11 (−14.7 ± 21.0; median −6.5; IQR −24.0–0.0) letters. Table 2B displays the number of eyes which experienced vision loss or gain between the onset of IOI and the last follow up. In 5 of 29 cases, we recorded a relevant delay between anterior and posterior segment manifestation.

The mean time from initial IOI symptoms and signs (ocular redness, pain, floaters, blurred vision, photophobia, flashing lights, decline in visual acuity, vitreous cells and/or hemorrhage, retinal vascular sheathing, filling defects and/or leakage, vasculitis, retinal arterial occlusion, retinal ischemia, and optic nerve inflammation) until any anti-inflammatory treatment (topical, intravitreal, oral, intravenous corticosteroid, and sub-Tenon’s capsule triamcinolone acetonide injections, as well as vitrectomy) was 3.3 ± 6.2 days (median 0, IQR 0–4.0, n = 41; Table 3). 

The time from the initial signs of IOI involving the posterior segment to anti-inflammatory treatment was reported in 37 out of 46 eyes (80.4%); among them, 13 eyes received immediate (same-day) treatment, while the treatment of 14 eyes was initiated within 1 to 7 days, while the remaining 10 were treated between 8 and 28 days. Accordingly, we could not evaluate the impact of time to treatment. Clinical manifestations (Supplemental Table S2) included anterior uveitis in 31/35 eyes (88.6%), vitreal infiltration in 36/39 (92.3%), retinal vascular occlusion in 29/34 (85.3%), retinal vasculitis in 33/39 (84.6%), retinal hemorrhages in 13/16 (81.3%), and macular involvement in 7/12 eyes (58.3%). There was no correlation between symptom duration and macular involvement in seven reported cases. 

Treatment initiation in response to intraocular inflammation (Supplemental Table S3) was reported with topical corticosteroids (CSs) in 33/34 eyes (94.1%), intraocular CSs in 5/8 (62.5%), peroral CSs in 19/30 (63.3%), and intravenous ± peroral CSs in 11/13 eyes (76.9%). Systemic antibiotics were administered in three cases; moreover, none of the patients received intravitreal antibiotics. Vitrectomy was performed in four (8.7%) eyes for different reasons; endophthalmitis was suspected in two (4.3%) eyes at baseline. No systematic treatment patterns were identified in response to intraocular inflammation. The first doses of intravenous and peroral corticosteroids were 516.0 ± 437.4 (median: 375, IQR: 70–1000, range: 40–1000) mg/day and 48.0 ± 19.7 (median: 50, IQR 30–60, range 20–100) mg/day, respectively. There was no difference in visual acuity at any time point between patients who received intravenous and peroral corticosteroids.

Visual outcomes did not differ between eyes treated with systemic (n = 29, 63.0%) and local (n = 7, 15.2%) corticosteroids; however, patients who received local corticosteroids had better visual outcomes 18–56 days after onset of IOI. This suggested that the intensity of treatment depended on the baseline severity of the inflammatory signs and vision loss. 

**Figure 2:**
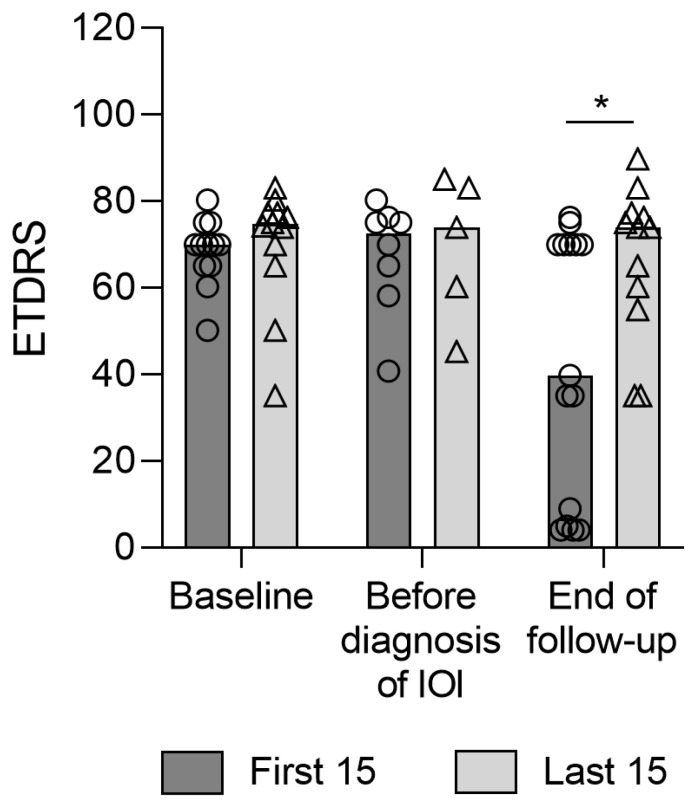
Evolution of visual acuity in the first and last 15 reported eyes from baseline (beginning of brolucizumab treatment) until end of follow up. Circles: Datapoints for the first 15 reported eyes; Triangles: Datapoints for the last 15 reported eyes; *: significant difference between the first 15 and the last 15 (*p* = 0.03).

The comparison of the 15 first and last reported cases indicated an improvement in the final visual outcomes following local and systemic corticosteroid treatments. The mean changes in visual acuity in the chronologically first- and last-treated patients were −25.3 ± 27.1 (median −23.1, IQR: −56 to 0; *p* = 0.006, effect size: r = 0.71) and −2.6 ± 7.3 (median 0, IQR: −8 to 0; *p* = 0148) ETDRS letters, respectively (*p* = 0.019, effect size: r = 0.63; Figure 2).

Furthermore, we assessed the potential impact of the number of intravitreal injections prior to the switch to brolucizumab treatment. We observed no difference in baseline BCVA and temporal changes in vision or in the time from brolucizumab initiation to IOI onset (<20 (5–18) injections before switch: 15.9 ± 9.5 (median 14.0, IQR: 8.0 to 21.0) days; >30 (32–94) injections before the switch: 16.5 ± 13.1 (median 11.0, IQR 7.0 to 27.0) days; *p* = 0.78).

## 4. Discussion

Given the extensive real-world reports on the therapeutic potential of brolucizumab to treat newly diagnosed and insufficiently responsive pre-treated nAMD [20,21,23,24,25,27,28,30,31,48,49], brolucizumab should be established in our armamentarium for nAMD therapy. However, it involves a considerable risk of IOI (≈4% (range 0–22%) [30,33,34,76,77]), with roughly 1% of the cases showing severe IOI [33,78]. Most of the reported cases presented a mild clinical picture without permanent vision loss and did not require systemic therapy [24,25,48]. Moreover, many series reported favorable outcomes even in severe cases following early diagnosis and therapy [22,32,33]. One recently published case series of IOI came from an Indian multicenter study and found a mild course requiring only topical corticosteroids in 38%, while the remaining 62% received a systemic treatment. All of these eyes responded well to a combination of topical and systemic corticosteroids but required a recovery time of three months to reach pre-IOI visual acuity; none of the eyes in this series encountered permanent severe vision loss [77]. This tenet has also been reported in a recent systematic review by Wykoff and colleagues [79]. Both papers strongly support our conclusion that with early diagnosis and qualified and timely treatment, severe and permanent vision loss is preventable in the vast majority of cases. 

Notably, only 31 papers referring to 47 eyes sufficiently detailed the outcomes of IOI involving posterior segments, which limited the analysis of several parameters, including baseline visual acuity, the presence of macular involvement, the severity of vitreal infiltration, vascular occlusion and vasculitis, the impact of time to treatment, and the treatment route. Nevertheless, we observed a period effect, which indicated progressive learning as demonstrated by mean losses of 25.3 and 3.2 letters in early and recent cases, respectively. This is suggestive of regained interest in brolucizumab at least in cases with high treatment demand [44]. Further, increasing recognition and awareness of brolucizumab-related IOI has improved the diagnostic sensitivity and functional outcomes, as indicated by the Novartis adverse event reporting database, with a retinal vasculitis (RV)/retinal vascular occlusion (RO) reporting rate of 7.5/10,000 injections and a related decline in severe vision loss from 5.9 to 4.1/10,000 injections [80]. IOI with RV/RO was reported in 2.1% of cases, with corresponding severe vision loss in 0.7% of the cases, in a post hoc analysis of HARRIER and HAWK data [38] as well as real-world studies [25,81]. This suggests that IOI involving the posterior segment is a rare event and does not necessarily cause severe vision loss. Our findings demonstrated the therapeutic potential of the early treatment of IOI with posterior segment involvement to improve outcomes [82]. This is consistent with a recent meta-analysis using a random-effects model including 14 randomized controlled trials on 6759 eyes, with no difference in the risk of severe sight-threatening IOI outcomes and retinal vascular occlusion between different anti-VEGF agents. Contrastingly, a six-fold higher incidence of IOI and a 1.6-fold higher incidence of vitreal opacifications was reported with brolucizumab than with aflibercept treatment [78]. 

In patients with IOI involving the posterior segment, significant vitreal infiltration may obscure the visualization of retinal details [50]. Therefore, wide-field angiography is generally recommended to determine the treatment strategy in the case of suspected IOI with RV/RO [44]. Surprisingly, wide-field angiography was not implemented in most cases to diagnose brolucizumab-induced IOI, which may have contributed to under- and misdiagnosis, as demonstrated by the three suspected endophthalmitis cases in our series. IOI is angiographically associated with retinal vasculitis in >70% of cases, with some of the cases progressing to central vascular occlusion and severe permanent vision loss [38]. In this meta-analysis, retinal vasculitis was reported in 33 (70%) eyes, which was associated with vascular occlusion in most cases (61.5% of all cases and 82.5% of cases with vascular occlusion). All four patients treated in our institution demonstrated a strong and long-lasting anti-leakage effect of brolucizumab despite severe IOI [39]. 

Since brolucizumab has only recently been approved for use in DME by the FDA, only one case of retinal arterial occlusion possibly related to brolucizumab has been reported [83], with no cases of DME in our series. Therefore, our findings cannot be readily extrapolated to patients with diabetes; however, this aspect has been addressed in the one-year safety findings obtained from KESTREL and KITE, where no new safety signals were observed [84].

This systematic literature search assessed all articles related to brolucizumab-induced IOI and its treatment outcomes on a single-patient basis. The reporting quality varied remarkably between the single case reports, which unescapably induces heterogeneity bias. For instance, the time from brolucizumab initiation until IOI was reported in 41/46 cases (89%), but the time from initial signs of IOI involving posterior segment to anti-inflammatory treatment was reported in only 37 out of 46 eyes (80.4%). Even visual acuity before the start of brolucizumab was not always reported (82.6%). The absence of such crucial information hindered addressing important points such as the impact of time to treatment. 

As indicated by the severity of baseline findings, this analysis of cases reporting treatment outcomes over time may have an inherent reporting bias given that they represent more spectacular cases. Therefore, the real-life outcomes might be even more favorable [80]. This reporting bias towards more severe cases was evidenced by the JBI Critical Appraisal Tool rating; however, the extent of this bias cannot be accurately estimated. Given the negative impact of insufficient disease control on long-term functional outcomes in nAMD [9,47] and the fact that up to 40% of eyes do not achieve disease stability with supportable treatment intervals [5], these cases still require attention from the medical community [24,33,76]. The careful selection of patients who accept the inherent chances and risks, thorough clinical examination prior to each injection in the first year and a minimal treatment interval of 8 weeks after the loading phase, appears to be fundamental for maximal risk mitigation [43]. Finally, given the small number of published cases and the short time of only two years since the publication of the first cases, we felt the registration of this meta-analysis would not be meaningful. 

## 5. Conclusions

In conclusion, our findings of a mean vision loss of 3.2 letters in recently reported cases and the downward trend of reported severe functional outcomes justify the use of brolucizumab from a benefit–risk perspective in cases with insufficient responses to other available and approved anti-VEGF drugs, prior to the establishment of systematic evidence or the development of comparably effective and durable new therapies.

## Figures and Tables

**Table 1:** Overview of included studies and published cases. n. r.: not reported; R: right eye; L: left eye.

Study Identifier	No. of Patients	Gender (0 = Male, 1 = Female)	Age (Years)	Patient Origin	No. of Eyes	Affected Eye	Treatment-Naïve Eyes (0 = No, 1 = Yes)
Angerer 2020 [53]	1	1	80	n. r.	1	R	0
Barchichat 2022 [54]	1	1	81	n. r.	2	R/L	0
Baumal 2020 [50]	3	1, 1, 1	74, 80, 71	n. r., n. r., n. r.	3	n. r., n. r., L	0, 0, 0
Bilgic 2021 [23]	1	1	71	n. r.	1	L	0
Fukuda 2021 [55]	1	1	77	n. r.	1	L	1
Giunta 2022 [25]	1	n. r.	n. r.	n. r.	1	n. r.	0
Haensli 2021 [22]	3	1, 0, 0	86, 84, 76	Swiss, Swiss, Swiss	3	R, L, L	0, 0, 0
Haug 2020 [56]	1	1	88	caucasian	2	R/L	0, 0
Hikichi 2021 [57]	3	0, 0, 1	72, 82, 94	Japanese	3	R, R, R	0, 0, 0
Iesato 2022 [58]	1	0	72	n. r.	1	R	n. r.
Ito 2022 [27]	2	0, 0	72, 63	Japanese, Japanese	2	n. r., n. r.	1, 1
Iyer 2020 [59]	1	1	76	Caucasian	1	R	0
Jain 2020 [60]	1	1	92	Caucasian	1	L	0
Kataoka 2021 [61]	3	1, 0, 0	52, 68, 71	Japanese, Japanese, Japanese	3	R, R, R	0, 0, 1
Kaupke 2021 [62]	1	n. r.	n. r.	n. r.	1	R	0
Kessler 2022 [63]	1	1	77	Caucasian	2	R/L	0
Kessler 2022 [64]	1	1	76	Caucasian	2	R/L	0
Kondapalli 2020 [65]	1	1	77	n. r.	1	R	0
Kusuhara 2022 [66]	1	1	75	n. r.	1	R	0
Leclaire 2022 [67]	1	0	77	n. r.	1	R	0
Lee 2022 [68]	1	0	71	n. r.	1	L	0
Montesel 2021 [21]	1	1	85	n. r.	1	n. r.	0
Narayanan 2021 [69]	1	1	62	n. r.	1	R	0
Nguyen 2022 [70]	1	0	73	Caucasian	1	R	0
Riedel 2021 [71]	1	1	78	n. r.	2	R/L	0
Rübsam 2022 [72]	1	n. r.	n. r.	n. r.	1	n. r.	1
Saito 2022 [73]	1	1	72	Japanese	1	R	0
Shigemoto 2021 [74]	1	0	71	Caucasian	1	R	0
Singer 2021 [35]	2	1, 0	79, 69	n. r., Asian	2	L, n. r.	1, 1
Witkin 2020 [36]	1	1	92	n. r.	1	R	0
Yoshikawa 2021 [75]	1	1	69	n. r.	1	R	0

**Table 2:** Evolution of visual acuity (VA). (**A**) Evolution of VA over time; (**B**) change in visual acuity after onset of intraocular inflammation.

(**A**)						
**VA (ETDRS Letters)**	**n**	**Mean**	**SD**	**Median**	**IQR (25–75%)**	**Range**
Baseline	44	65.6	17.6	69.9	60–76	20–94
Maximum	17	67.6	15.3	73.9	59–79	35–85
Last	43	50.8	30.5	63.4	15–75	1–94
(**B**)						
		**n (Finding Reported)**		**% of Reported Eyes (n/N)**	% of all Eyes (n = 41) *	
Visual loss >15 letters		16		39.0	34.8	
Visual loss 6–15 letters		6		14.6	13.0	
Stable vision (−5 to +5 letters)		16		39.0	34.8	
Visual gain 6–15 letters		3		7.3	6.5	

n, number of observations; SD, standard deviation; IQR, interquartile range. Information about visual acuity at baseline was missing for two eyes. Maximum: maximum VA during brolucizumab treatment; * visual acuity was not reported for two eyes at baseline and for 3 at follow-up.

**Table 3:** Time interval between last intravitreal brolucizumab injection and the development of intraocular inflammation.

Time (Days) between	n	Mean	SD	Median	IQR (25–75%)	Range
last brolucizumab until first signs of anterior uveitis	30	15.3	12.8	13.5	6.5–21.3	0–56
last brolucizumab until first signs of posterior uveitis	44	19.5	16.9	14.0	7.3–27.0	0–84
last brolucizumab until diagnosis of posterior uveitis	4	22.3	17.6	21.0	8.0–29.5	1–84

## Data Availability

Data of this meta-analysis will be made available on qualified request to the corresponding author.

## Supplementary Materials

The following supporting information can be downloaded at: https://www.mdpi.com/article/10.3390/jcm12144671/s1. Supplemental Table S1: JBI Critical Appraisal Tool Rating; Supplemental Table S2: Clinical presentation of intraocular inflammation in response to intravitreal brolucizumab injection; Supplemental Table S3: Treatment initiation in response to intraocular inflammation.
