# Supplementary material for: Functional Outcomes of Brolucizumab-Induced Intraocular Inflammation Involving the Posterior Segment—A Meta-Analysis and Systematic Review
Source: J Clin Med. 2023 Jul 14;12(14):4671. doi: 10.3390/jcm12144671 (PMC10380786; doi:10.3390/jcm12144671)
Supplement: Supplementary file 1 [file jcm-12-04671-s001.zip › jcm-2421788-supplementary.pdf]

Supplementary Materials

Search Strings

PubMed
((Brolucizumab[Title/Abstract] OR Beovu[Title/Abstract]) AND (AMD[Title/Abstract] OR macular degeneration[Title/Abstract] OR macula*[Title/Abstract] AND ((inflammation[Title/Abstract] OR vasculitis[Title/Abstract] OR occlusion[Title/Abstract] OR uveitis[Title/Abstract]))

Scopus
TITLE-ABS-KEY((Brolucizumab OR Beovu) AND (AMD OR macular degeneration OR macula*) AND (inflammation OR vasculitis OR occlusion OR uveitis))

ClinicalTrials.gov
Condition or disease: wet age-related macular degeneration; Intervention/treatment: Brolucizumab

CENTRAL
Brolucizumab OR Beovu in Title Abstract Keyword AND AMD OR macular degeneration OR macula* OR inflammation OR vasculitis OR occlusion OR uveitis in Title Abstract Keyword

Supplemental Table S1: JBI Critical Appraisal Tool Rating

Paper	1. Were there clear criteria for inclusion in the case series?	2. Was the condition measured in a standard, reliable way for all participants included in the case series?	3. Were valid methods used for identification of the condition for all participants included in the case series?	4. Did the case series have consecutive inclusion of participants?	5. Did the case series have complete inclusion of participants?	6.Was there clear reporting of the demographics of the participants in the study?	7. Was there clear reporting of clinical information of the participants?	8. Were the outcomes or follow-up results of cases clearly reported?	9. Was there clear reporting of the presenting sites'/clinics' demographic information?	10. Was statistical analysis appropriate?
Jain 2020	1	n.a.	1	n.a.	0	1	1	1	0	n.a.
Saito 2022	1	n.a.	1	n.a.	0	1	0	1	0	n.a.
Haug 2020	1	n.a.	1	n.a.	0	1	1	1	0	n.a.
Iyer 2020	1	n.a.	1	n.a.	0	1	0	1	1	n.a.
Kusuhara 2022	1	n.a.	1	n.a.	0	1	1	1	1	n.a.
Singer 2021	1	1	1	1	1	1	1	1	1	1
Yoshikawa 2021	1	n.a.	1	n.a.	0	1	0	1	1	n.a.
Rübsam 2022	1	1	1	1	n.k.	1	0	1	0	1
Fukuda 2021	1	1	1	1	1	1	0	0	0	1
Montesel 2021	1	1	1	1	1	1	0	1	1	1
Narayanan 2021	1	n.a.	1	n.a.	0	1	0	0	1	n.a.
Hänsli 2021	1	1	1	1	1	1	1	1	0	1
Bilgic 2021	1	1	1	1	1	1	0	1	1	1
Shigemoto 2021	1	n.a.	1	n.a.	0	1	0	1	1	n.a.
Giunta 2022	1	1	1	1	1	1	1	1	1	1
Kessler 2022	1	n.a.	1	n.a.	0	1	0	1	0	n.a.
Leclaire 2022	1	n.a.	1	n.a.	0	1	0	1	0	n.a.
Barchichat 2022	1	n.a.	1	n.a.	0	1	1	1	1	n.a.
Lee 2022	1	n.a.	1	n.a.	0	1	1	1	1	n.a.
Ito 2022	1	1	1	1	n.k.	1	0	1	1	n.a.
Iesato 2022	1	n.a.	1	n.a.	0	1	0	1	0	n.a.
Hikichi 2021	1	1	1	n.k.	0	1	1	1	1	n.a.
Nguyen 2022	1	1	n.k.	n.k.	0	1	0	0	1	n.a.
Witkin 2020	1	1	1	n.k.	n.k.	1	1	1	1	n.a.
Kataoka 2021	1	1	1	n.k.	0	1	1	1	1	n.a.
Kondapalli 2020	1	n.a.	1	n.a.	0	1	0	0	0	n.a.
Riedel 2021	1	n.a.	1	n.a.	0	1	0	0	0	n.a.
Kaupke 2021	1	n.a.	1	n.a.	0	1	1	1	0	n.a.

Angerer 2020	1	n.a.	1	n.a.	0	1	0	0	0	n.a.
Kessler 2022 (2)	1	n.a.	1	n.a.	0	1	1	1	0	n.a.
Baumal 2020	1	1	1	1	n.k.	1	1	1	1	1

Note: 1 = yes; 0 = no; n.a. = not applicable; n.k. = not known.

Supplemental Table S2. Clinical presentation of intraocular inflammation in response to intravitreal Brolucizumab injection.

**Clinical finding**	**n (finding present)**	**% of reported eyes (n/N)**	**N total (reported)**	**% of all eyes (n=46)***
anterior uveitis	31	88.6	35	67.4
vitreal infiltration	36	92.3	39	78.3
retinal vascular occlusion	29	85.3	34	63.0
retinal vasculitis	33	84.6	39	71.7
retinal hemorrhages	13	81.3	16	28.3
macular involvement	7	58.3	12	15.2

* The portion of the total number of eyes is provided based on the assumption that findings not reported at diagnosis were not present at baseline

Supplemental Table S3. Treatment initiation in response to intraocular inflammation

	n (present)	% of reported eyes (n/N)	N total (reported)	% of all eyes (n=46)*
Corticosteroids:				
- topical	32	94.1	34	69.6
- intraocular	5	62.5	8	10.9
- peroral	19	63.3	30	41.3
- intravenous ± peroral	10	76.9	13	21.7
Systemic antibiotics	2	40	5	4.3
Hemodilutive agents	1	16.7	5	2.2
Vitrectomy	4	40	9	8.7

* The portion of the total population is provided based on the assumption that treatments not reported at diagnosis were not given.
